# Evaluation of the Diagnostic Accuracy of Different Imaging Modalities in Patients with Pathologic Nipple Discharge

**DOI:** 10.3390/diagnostics16213530

**Published:** 2026-10-27

**Authors:** Riku Togawa, Dina Batarseh, Helena Dahm, Thomas M. Deutsch, André Hennigs, Andrea Lemke, André Pfob, Walid Shaalan, Anne Stieber, Oliver Zivanovic, Jörg Heil, Michael Golatta, Fabian Riedel

**Affiliations:** 1 Breast Unit, Department of Obstetrics and Gynecology, Heidelberg University Hospital, Im Neuenheimer Feld 440, 69120 Heidelberg, Germany; dina.batarseh@med.uni-heidelberg.de (D.B.); fabian.riedel@med.uni-heidelberg.de (F.R.); 2 Breast Unit, Sankt Elisabeth Hospital, Max-Reger-Strasse 5–8, 69121 Heidelberg, Germany; 3 Department of Diagnostic and Interventional Radiology, Heidelberg University Hospital, Im Neuenheimer Feld 440, 69120 Heidelberg, Germany

**Keywords:** pathologic nipple discharge, breast cancer, ultrasound, mammography, ductography, MRI

## Abstract

**Background/Objectives:** To evaluate the diagnostic accuracy of different imaging modalities in patients with pathologic nipple discharge. **Methods:** This is a single-centre retrospective analysis of patients with pathologic nipple discharge. All performed imaging modalities were reviewed for their sensitivity and specificity in correctly diagnosing malignancy, as well as lesions with uncertain malignant potential (B3). **Results:** Of 388 patients, pathology or follow-up confirmed 41 cases of malignancy (15 breast cancer, 26 ductal carcinoma in situ (DCIS)), 151 cases of B3 lesions, and 196 benign cases. The combination of ultrasound and mammography/tomosynthesis achieved a sensitivity of 82.5% (95% CI 68.1–91.3) and a specificity of 83.5% (77.4–88.2) for the detection of malignancy. For the classification of B3 lesions as suspicious (BI-RADS 4/5), the sensitivity and specificity were 33.8% (26.7–41.7) and 83.5% (77.4–88.2), respectively. Ductography demonstrated a sensitivity of 89.3% (72.8–96.3) and a specificity of 13.3% (8.2–20.8) for malignancy, while MRI showed a sensitivity of 71.4% (45.4–88.3) and a specificity of 90.0% (76.9–96.0). In classifying B3 lesions as suspicious, ductography reached a sensitivity of 94.8% (89.7–97.5) and a specificity of 13.3% (8.2–20.8), compared to 75.9% (57.9–87.8) and 90.0% (76.9–96.0), respectively, for MRI. **Conclusions:** While the combination of ultrasound and mammography/tomosynthesis correctly classified 82.5% of breast malignancies associated with pathologic nipple discharge as suspicious, their sensitivity for classifying B3 lesions as suspicious was only moderate (33.8%). In patients with negative or inconclusive conventional imaging but persistent clinical suspicion, MRI may be considered as an additional diagnostic tool, whereas ductography, given its low specificity, is of limited value for assessing lesion dignity and may primarily serve to localize the affected duct prior to surgery. Further research is needed to determine which diagnostic modality provides the greatest benefit in specific cases.

## 1. Introduction

Nipple discharge (ND) is a common cause for presentation to a breast unit, as more than 80% of women experience ND during their lifetime [1,2,3]. A physiological ND is usually characterized by the discharge of yellowish-milky or greenish fluid from both nipples from several ducts. The reason is often fibrocystic breast disease, galactophore duct ectasia, or mastitis [4,5]. According to the 2022 American College of Radiology (ACR) Appropriateness Criteria, pathological nipple discharge (PND) is characterized by the presence of at least one of the following features: spontaneous discharge from a single duct orifice, unilateral discharge, serous discharge, or bloody discharge [4,5,6,7]. PND is of clinical significance due to its association with malignant breast lesions, although it is mostly caused by benign breast lesions such as intraductal papilloma (IDP), papillomatosis, or ductal ectasia [4,7,8,9,10,11,12]. ND has been reported as the presenting symptom in up to 7% of patients undergoing breast surgery [13].

Several diagnostic options are currently available to clarify the causes of PND. The Appropriateness Criteria published by the American College of Radiology (ACR) provide recommendations for the initial imaging evaluation of ND based on the patient’s age and gender [5,14]. Mammography and ultrasound represent the mainstay of the initial diagnostic workup. Depending on the clinical presentation and the findings of the initial assessment, additional diagnostic procedures, including ductography, magnetic resonance imaging (MRI), discharge cytology, and duct excision, may be considered in selected patients. However, there are no evidence-based standardized diagnostic algorithms, and considerable variability in the diagnostic workup exists between different breast units.

Although being the first diagnostic option in women older than 40 years, mammography alone has a low sensitivity (7–26%) in the detection of retroareolar lesions that are often small, intraductal, and without calcifications [3,4,15,16]. A negative mammography therefore does not exclude a secondary PND due to a breast lesion [3,15,17].

Ultrasound shows higher sensitivity (63–100%) in the evaluation of ductal structures and intraductal lesions that cause ND [2,9,16].

A recent study by Chung et al. demonstrated that the combination of ultrasound and mammography detected up to 93% of malignancies associated with PND with a negative predictive value of 98% [18].

When PND can be demonstrated at the time of diagnostic checkup, ductography can be performed. Ductography can delineate ductal abnormalities, including irregularities, stenoses, distortion, and ectasia; however, examination failure rates of up to 15% and limited differentiation between benign and malignant lesions remain important limitations. Sensitivity of ductography is higher compared to conventional mammography but it goes along with additional radiation exposure and is less specific than MRI [1,19,20]. MRI has shown a higher diagnostic sensitivity than ductography, with reported sensitivity rates of up to 96% [21,22].

Ductoscopy is another imaging modality in patients with PND to visualize the mammary duct from which the discharge originates using an endoscopy device, with which the inner duct can be visualized [23,24]. Although its sensitivity is high (94%) it is available in only few breast units [1].

ND cytology is of limited diagnostic accuracy with a false negative rate of nearly 50% [25,26].

In this monocentric retrospective analysis, we assessed the diagnostic accuracy of mammography and ultrasound in the evaluation of PND and evaluated the ability of additional diagnostic modalities, including ductography, cytology, and MRI, to differentiate between benign and malignant etiologies of PND. By analyzing a large real-world cohort encompassing malignant, B3, and benign lesions over an 11-year period, we aimed to complement existing evidence on the diagnostic work-up of PND, with particular emphasis on the diagnostic limitations of conventional imaging and the potential complementary value of additional diagnostic modalities.

## 2. Materials and Methods

### 2.1. Study Design and Patients

This 11-year retrospective study was conducted at a certified high-volume breast unit to present data from routine clinical practice, evaluating the clinical, radiological, and pathological findings of patients presenting with PND between 2012 and 2022. The study was approved by the institutional review board (S-616/2024). The need for informed consent was waived. This study was reported in accordance with the STROBE guidelines for observational studies and the STARD 2015 guidelines for diagnostic accuracy studies; both completed checklists are provided as Supplementary Material.

The electronic health record was reviewed by two gynecologists specialized in breast diagnostics. The clinical history, breast imaging findings and assessments, biopsy methods, pathology, and follow up data were recorded.

PND was defined according to the 2022 ACR Appropriateness Criteria as discharge demonstrating at least one of the following features: spontaneous discharge from a single-duct orifice, unilateral discharge, or serous or bloodstained discharge.

Non-spontaneous discharge was defined as secretion elicited by mechanical pressure on the breast or nipple, either by the patient (e.g., during skin care, showering, or physical activity) or by manual compression during clinical examination. If discharge was not reproducible during clinical examination, its occurrence and characteristics were assessed from the documented patient history and, when available, supporting evidence such as patient-provided photographs or visible staining of clothing. Classification as PND required documentation of at least one of the predefined ACR-based characteristics. Patients without any of these characteristics or with insufficient documentation to establish PND were excluded. Missing information on individual discharge characteristics did not result in exclusion if at least one other ACR-defined PND feature was documented. Patients were excluded due to insufficient documentation only when none of the defining PND characteristics could be established from the available records.

### 2.2. Diagnostic Workup

Standard imaging protocol for patients presenting with PND consisted of digital breast mammography and tomosynthesis followed by ultrasound in women older than 40 years. At our institution, mammographic imaging generally comprised a medio-lateral-oblique (MLO) view acquired using digital breast tomosynthesis and a conventional digital mammographic cranio-caudal (CC) view. However, when recent and diagnostically adequate mammographic examinations from the breast screening program or external institutions were available, mammography was not routinely repeated. Tomosynthesis was considered part of the mammographic examination and was not analyzed as a separate imaging modality. Younger women received breast ultrasound as the initial examination, followed by mammography and/or tomosynthesis when considered clinically indicated by the performing physician. If the PND was reproducible at the time of examination discharge cytology was also performed. Further examinations such as ductography and MRI were performed secondarily. Breast MRI examinations were performed at 1.5 T using the institutional standard breast MRI protocol, including diffusion-weighted imaging and contrast-enhanced fat-suppressed T1-weighted sequences. MRI examinations were interpreted by a radiologist with more than 20 years of experience in breast imaging. Each imaging modality was interpreted according to the current edition of Breast Imaging Reporting and Data System (BI-RADS). A positive examination was defined as BI-RADS 4 or 5 with a recommendation for biopsy, whereas a negative examination was defined as BI-RADS 1, 2 or 3. In the initial diagnostic work-up, BI-RADS 0 was assigned in 7 ultrasound examinations, 12 mammography/tomosynthesis examinations, and 2 MRI examinations. As BI-RADS 0 represents an incomplete assessment and cannot be classified as either positive or negative, these examinations were retrospectively reassessed by one of the two gynecologists specialized in breast diagnostics, blinded to the final histopathological diagnosis or follow-up outcome and a definitive BI-RADS category was assigned. Consequently, no BI-RADS 0 examinations remained for the statistical analysis. Regarding histopathology, patients were classified according to the final available histopathological diagnosis. When both biopsy and subsequent surgical excision were performed, the diagnosis obtained from the surgical specimen was considered definitive and superseded the preceding biopsy diagnosis. As no dedicated BI-RADS lexicon exists for ductography, examinations were classified as positive or negative based on the presence of ductal abnormalities. Positive findings included intraductal filling defects, ductal obstruction or abrupt cut-off, and suspicious ductal irregularities. Examinations without these findings were considered negative if adequate visualization of the ductal system was achieved. Technically non-diagnostic examinations, including unsuccessful duct cannulation, contrast extravasation, or inadequate ductal opacification, were excluded from the diagnostic performance analysis rather than classified as negative.

### 2.3. Statistical Analysis

The study cohort was described by the measures of empirical distribution. Depending on the level of measurement, mean and standard deviation as well as absolute and relative frequencies were calculated. Patients with missing essential baseline information required for study eligibility or classification were excluded from the analysis. For remaining missing data affecting individual variables or diagnostic procedures, an available-case approach was used, with exclusion limited to the respective analysis. No imputation of missing data was performed. The sensitivity, specificity, positive predictive value (PPV), negative predictive value (NPV) were calculated for each of the different imaging modalities. For sensitivity, specificity, PPV, and NPV, 95% confidence intervals were calculated using the Wilson score method for binomial proportions. Cytology results reported as indeterminate could not be classified as positive or negative and were therefore excluded from the cytology analysis.

Malignancy was defined as malignant lesions, including invasive breast cancer and ductal carcinoma in situ (DCIS). Lesions of uncertain malignant potential (B3) included atypical ductal hyperplasia (ADH), atypical lobular hyperplasia (ALH), lobular carcinoma in situ (LCIS), and other atypical proliferations, with or without associated papilloma, as diagnosed by histopathology [27]. The benign group comprised all benign findings, as well as cases without evidence of malignancy after a minimum follow-up period of 24 months. Diagnostic performance was assessed separately for malignant and B3 lesions. For the analysis of malignancy, malignant lesions were compared with benign lesions, with B3 lesions excluded. For the analysis of B3 lesions, B3 lesions were compared with benign lesions, with malignant lesions excluded.

Statistical analyses were performed using R (version 4.1.0; R Foundation for Statistical Computing, Vienna, Austria) and RStudio (version 1.0.136; RStudio, Inc., Boston, MA, USA).

## 3. Results

### 3.1. Study Cohort

**Figure 1:**
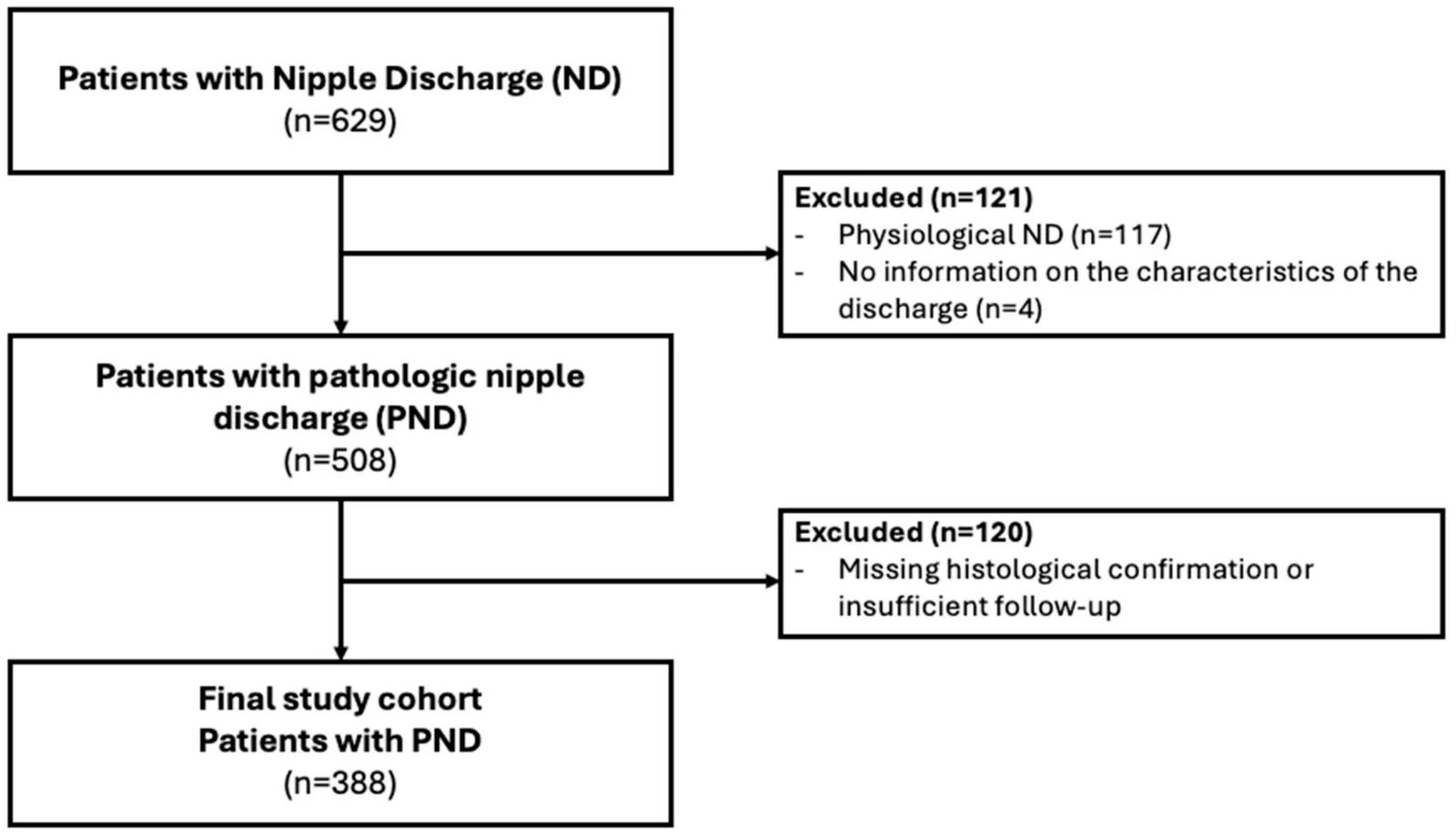
Flowchart of patient selection.

During the study period, 629 patients presenting with ND were retrospectively identified. Of these, 121 patients were excluded because they presented with physiological ND or because the available documentation did not provide sufficient information on the characteristics of the secretion to classify it as pathological. The remaining 508 patients fulfilled the criteria for PND, as defined in Section 2.1 according to the 2022 ACR Appropriateness Criteria. Of these, 120 patients were excluded due to missing histological confirmation and insufficient follow-up. Thus, 388 patients with PND and either confirmed histology (biopsy or ductectomy) or a follow-up of at least 24 months were eligible for further analysis (Figure 1).

The final study cohort comprised 41 cases of malignancy (15 breast cancer, 26 ductal carcinoma in situ), 151 cases of B3 lesions including 141 cases of intraductal papilloma, 104 other benign pathologies, and 92 cases classified as benign based on the absence of malignancy during a follow-up period of at least 24 months. The mean duration of follow-up was 45.4 ± 24.8 months (min. 24 months, max. 120 months).

The mean age of the study cohort was 51.4 ± 13.9 years. In terms of laterality, ND was unilateral in 74.7% (*n* = 290) and bilateral in 25.3% (*n* = 98). Spontaneous discharge was observed in 56.2% (*n* = 218), while 40.0% (*n* = 155) presented with non-spontaneous discharge; in 3.8% (*n* = 15), the type of discharge was unknown. Regarding the nature of the discharge, it was bloody in 61.9% (*n* = 240), clear in 29.4% (*n* = 114), and milky in 5.4% (*n* = 21); in 3.3% (*n* = 13) of cases, the nature of the discharge was not specified. These data are summarized in Table 1.

### 3.2. Benign Lesions

Of all 388 cases, there were 196 benign cases. 104 patients underwent histological confirmation with core needle biopsy (8 cases, 7.7%), stereotactic vacuum biopsy (3 cases, 2.9%), ductectomy (92 cases, 88.5%) or MRI vacuum biopsy (1 case, 1.0%). Ninety-two cases were considered benign because of the absence of malignancy after ≥24 months of follow-up. The mean age of these patients was 48.6 ± 13.7 years. All patients received ultrasound examination, while 183 patients received additional mammography/tomosynthesis. MRI or ductography were performed in 141 cases. Ultrasound identified 178 cases as benign (BI-RADS ≤ 2), four cases were categorized as BI-RADS 3 and 14 cases as BI-RADS 4. Mammography/tomosynthesis identified 166 cases as benign (BI-RADS ≤ 2), zero cases as BI-RADS 3 and 17 cases as BI-RADS 4. A breakdown of the cases including histology, if a biopsy has been performed, is summarized in Table 2.

### 3.3. Malignant Lesions and Lesions with Uncertain Malignant Potential

Of all 388 cases, there were 41 cases of malignancies comprising 26 cases of DCIS and 15 invasive breast cancer. The mean age of the patients with malignant lesions was 63.0 ± 13.1 years. Histological confirmation was performed via core needle biopsy in 23 (56.1%) cases, stereotactic vacuum biopsy in 8 (19.5%) cases, ductectomy in nine (22.0%) cases and surgical biopsy in one (2.4%) case. Thirty-four (82.9%) cases of malignant lesions were identified by ultrasound and/or mammography and tomosynthesis alone, while seven (17.1%) cases were identified by additional MRI/ductography. Five (12.2%) cases were identified by ductography, one (2.4%) case by MRI, and one (2.4%) case was identified by both ductography and MRI.

Additionally, there were 151 cases with lesions of uncertain malignant potential comprising 141 cases of IDP, of which four (2.8%) were associated with atypia and 137 (97.2%) were without atypia, three cases of ADH, two cases of a radial scar, two cases of nipple adenoma, one case of an intraductal phyllodes tumor, one case of an atypical lobular hyperplasia (ALH), and one case of lobular carcinoma in situ (LCIS). The mean age of the patients was 51.6 ± 13.2 years. Histological confirmation was performed via core needle biopsy in 25 (16.6%) cases, stereotactic vacuum biopsy in three (2.0%) cases, ductectomy in 119 (78.8%) cases, surgical biopsy in one (0.7%) case, punch biopsy in two (1.3%) cases, and in one (0.7%) case a discharged tissue fragment was examined.

Among the 151 B3 lesions, 49 (32.4%) were classified as suspicious (BI-RADS 4) by ultrasound and 21 (13.9%) by mammography/tomosynthesis. Of these, 20 lesions were classified as BI-RADS 4 by both modalities, while 29 were classified as suspicious exclusively by ultrasound and one exclusively by mammography/tomosynthesis. Thus, a total of 50 patients (33.1%) received a suspicious BI-RADS 4 classification in at least one of the two modalities. Additional imaging yielded suspicious findings in 95 patients (62.9%), including 82 patients (54.3%) with findings on ductography only, five on MRI only (3.3%), and eight on both modalities (5.3%). Three patients did not undergo mammography/tomosynthesis and were therefore excluded from the combined diagnostic performance analysis, resulting in 148 evaluable B3 cases.

In the basic diagnostic work-up, consisting of the combination of ultrasound and mammography/tomosynthesis (*n* = 40 malignancies with complete imaging; one patient declined mammography), overall sensitivity for malignancy was 82.5% (95% CI 68.1–91.3), specificity 83.5% (77.4–88.2), PPV 52.4% (40.3–64.2), and NPV 95.6% (91.2–97.9). For invasive malignancy (*n* = 14), sensitivity was 92.9% (68.5–98.7), specificity 83.5% (77.4–88.2), PPV 30.2% (18.6–45.1), and NPV 99.3% (96.4–99.9); for DCIS (*n* = 26), sensitivity was 76.9% (57.9–89.0), specificity 83.5% (77.4–88.2), PPV 40.0% (27.6–53.8), and NPV 96.2% (92.0–98.2). For B3 lesions (*n* = 148; three patients declined mammography), sensitivity was 33.8% (26.7–41.7), specificity 83.5% (77.4–88.2), PPV 62.5% (51.5–72.3), and NPV 60.8% (54.6–66.6) (Table 3). Imaging findings of the B3 lesions other than intraductal papillomas without atypia are summarized in Table 4.

Among the 40 patients with malignancy included in the basic diagnostic work-up analysis, six had a documented palpable finding for which an association with the subsequently diagnosed malignancy could not be excluded on retrospective review. After exclusion of these patients, sensitivity remained similar (79.4% vs. 82.5% in the primary analysis), while specificity remained unchanged at 83.5%; PPV and NPV were likewise not materially altered. Detailed results are provided as Supplementary Material.

Of the 41 patients with malignancy, 28 (68.3%) had an evaluable ductography as part of their extended diagnostic work-up, compared with 135 patients (89.4%) in the B3 lesion cohort. In the malignancy group, ductography was attempted in 33 patients, of whom 28 (84.8%) had technically successful examinations, including 9 patients with invasive carcinoma and 19 with DCIS. Five examinations were non-diagnostic due to unsuccessful duct cannulation (*n* = 2) or contrast extravasation (*n* = 3). In these patients ductography demonstrated a sensitivity of 89.3% (95% CI 72.8–96.3), specificity of 13.3% (8.2–20.8), PPV of 20.3% (14.2–28.3), and NPV of 83.3% (60.8–94.2). When analysed separately, the invasive breast cancer subgroup (*n* = 9) showed a sensitivity of 66.7% (35.4–87.9), specificity of 13.3% (8.2–20.8), PPV of 5.8% (2.7–12.0), and NPV of 83.3% (60.8–94.2), whereas the DCIS subgroup (*n* = 19) achieved a sensitivity of 100% (83.2–100), specificity of 13.3% (8.2–20.8), PPV of 16.2% (10.6–24.0), and NPV of 100% (79.6–100). In the B3 lesion cohort, ductography was attempted in 143 patients, of whom 135 (94.4%) had technically successful examinations. Eight examinations were non-diagnostic due to unsuccessful duct cannulation (*n* = 5), contrast extravasation (*n* = 2), or failure of ductal opacification despite successful cannulation (*n* = 1). In this cohort ductography yielded a sensitivity of 94.8% (89.7–97.5), specificity of 13.3% (8.2–20.8), PPV of 56.6% (50.1–62.9), and NPV of 68.2% (47.3–83.6) (Table 5).

Fourteen (34%) patients with malignancy and 29 (19%) patients with B3 lesions received MRI as part of their extended diagnostic work-up. In the malignancy group, MRI demonstrated a sensitivity of 71.4% (95% CI 45.4–88.3), specificity of 90.0% (76.9–96.0), PPV of 71.4% (45.4–88.3), and NPV of 90.0% (76.9–96.0). When analysed separately, the invasive breast cancer subgroup (*n* = 7) showed a sensitivity of 57.1% (25.0–84.2), specificity of 90.0% (76.9–96.0), PPV of 50.0% (21.5–78.5), and NPV of 92.3% (79.7–97.3), whereas the DCIS subgroup (*n* = 7) achieved a sensitivity of 85.7% (48.7–97.4), specificity of 90.0% (76.9–96.0), PPV of 60.0% (31.3–83.2), and NPV of 97.3% (86.2–99.5). In the B3 cohort MRI yielded a sensitivity of 75.9% (57.9–87.8), specificity of 90.0% (76.9–96.0), PPV of 84.6% (66.5–93.8), and NPV of 83.7% (70.0–91.9) (Table 6).

Discharge cytology was performed in 31 of the 41 malignant cases; two cases with indeterminate cytology were excluded, leaving 29 evaluable cases. In seven cases cytology was diagnosed as positive, while in the other 22 cases cytology was negative. This resulted in a sensitivity of 24.1% (95% CI 12.2–42.1), a specificity of 98.7% (95.4–99.6), a PPV of 77.8% (45.3–93.7) and an NPV of 87.3% (81.5–91.5). In the B3 cohort, discharge cytology was performed in 132 of the 151 cases, of which 130 yielded an evaluable result, with a sensitivity of 8.5% (4.8–14.5), specificity of 98.7% (95.4–99.6), a PPV of 84.6% (57.8–95.7), and an NPV of 55.9% (50.0–61.7) (Table 7).

## 4. Discussion

In this retrospective single-centre series of 388 patients with PND it is demonstrated that the standard diagnostic approach—combining ultrasound and mammography/tomosynthesis—provides high sensitivity (82.5%; 95% CI 68.1–91.3) and specificity (83.5%; 77.4–88.2) for detecting malignancy.

Regarding the detection of malignancy, our findings are consistent with previous reports demonstrating a high diagnostic value of combined mammography and ultrasound in patients with PND [4,5,22,25,28]. In our cohort, conventional imaging identified the majority of malignant lesions, with a sensitivity of 82.5%, supporting its central role in the initial diagnostic evaluation of PND. Nevertheless, seven of 41 malignancies were detected only by additional MRI and/or ductography, indicating that a residual risk of malignancy remains after negative conventional imaging and that further diagnostic evaluation may be appropriate in selected patients.

While conventional imaging thus demonstrated good diagnostic performance for malignant lesions, its sensitivity was considerably lower for lesions of uncertain malignant potential. In this cohort, the sensitivity for classifying B3 lesions as suspicious (BI-RADS 4/5) was only 33.8% (95% CI 26.7–41.7), with a specificity of 83.5% (77.4–88.2), indicating that a large proportion of B3 lesions were not classified as suspicious by ultrasound and mammography alone. This is aligned with known limitations: B3 lesions often lack distinctive imaging features and are typically small, retroareolar, or non-calcified, making detection challenging on standard modalities [29,30,31].

Accurate detection of B3 lesions remains clinically important, although these lesions are not malignant per se, as they may be associated with a risk of malignant upgrade to DCIS or low-grade invasive carcinoma. Importantly, the magnitude of this risk varies considerably according to histological subtype. While upgrade rates of up to approximately 35% have been reported for higher-risk B3 entities such as ADH, lower but still clinically relevant rates are reported for intraductal papillomas, ranging from 6–10% in lesions without atypia to 21–29% in those with atypia [32,33,34,35,36,37,38]. This distinction is particularly relevant to our cohort, in which intraductal papillomas without atypia represented the vast majority of B3 lesions. Therefore, underdiagnosing or overlooking these lesions carries potential oncologic risk. High-risk lesions such as ADH or lobular neoplasia are considered not only potential precursors of malignancy but also risk indicators for future malignancy in both the ipsilateral and contralateral breast [39,40]. Given the limitations of conventional imaging, further diagnostic work-up is warranted if no lesion is detected in women with PND.

In the present cohort, ductography demonstrated the highest sensitivity (89.3%; 95% CI 72.8–96.3) for malignancy detection and was particularly effective for identifying B3 lesions (sensitivity 94.8%; 89.7–97.5). However, its specificity was extremely low at 13.3% (8.2–20.8), indicating a substantial rate of false-positive results. These findings align with prior reports that emphasize ductography’s high sensitivity but limited specificity, especially when used without correlation to clinical or adjunct imaging findings. Several studies have highlighted the limited ability of ductography to distinguish between benign, atypical, and malignant ductal abnormalities, due to overlapping morphologic features such as ductal wall irregularities or filling defects [41,42].

The diagnostic yield of ductography is also highly operator-dependent and technically limited in patients with non-active discharge or ductal obstruction [43].

A 2018 study by Istomin et al. reported a specificity of 76% for ductography; however, reported specificity varies considerably across the literature, with some studies reporting values as low as 6–40%, depending on patient selection and interpretation criteria [5,41,44]. Similarly, a systematic review by Berger et al. found that, despite its high sensitivity, the low specificity of ductography limits its value as a standalone modality, as positive findings frequently lead to further diagnostic procedures and may ultimately result in unnecessary surgery for benign lesions [6]. Consequently, ductography appears to have limited value for determining lesion dignity, while its clinical utility may lie primarily in the visualization and localization of the affected ductal system, particularly when planning subsequent surgical management.

The emergence of digital breast tomosynthesis (DBT)-assisted ductography and contrast-enhanced ductal imaging may improve specificity, but these techniques are not yet widely available or standardized in clinical workflows [45,46,47].

MRI, on the other hand, displayed a more balanced profile: it showed a high specificity (90.0%; 95% CI 76.9–96.0) for malignancy but a sensitivity of 71.4% (45.4–88.3). This sensitivity is lower than previously reported values of approximately 85% to 100%, while reported specificity ranges from 37% to 97%, influenced by lesion morphology or enhancement kinetics [5,6,28,48]. However, MRI was performed in a small and highly selected subgroup, including only 14 patients with malignant lesions. Thus, the observed sensitivity of 71.4% was based on 10 of 14 correctly identified malignancies and should be interpreted with caution due to the resulting statistical uncertainty and potential referral bias. For B3 lesions, MRI in our cohort reached a sensitivity of 75.9% (57.9–87.8) and specificity of 90.0% (76.9–96.0).

The 2017 meta-analysis by Berger et al. reported superior sensitivity (92% vs. ~69%) and specificity for MRI versus ductography in detecting any breast lesion in patients with ND [6]. Nonetheless, ductography can still provide valuable ductal mapping, particularly when standard imaging and MRI are unavailable or inconclusive [6,41,46,49,50].

Cytology demonstrated a low sensitivity for the detection of malignancy (24.1%; 95% CI 12.2–42.1) and particularly for B3 lesions (8.5%; 4.8–14.5), despite a high specificity in both categories (98.7%; 95.4–99.6). These findings are consistent with multiple previous studies reporting that cytology from ND has limited diagnostic sensitivity, often ranging between 11–34% for malignancy detection, but maintains a high specificity exceeding 90% when malignant cells are identified [26,51].

While cytology may serve as a low-cost adjunctive tool, it should not be used as a stand-alone diagnostic method for PND.

This study has several limitations that must be acknowledged. Firstly, the retrospective design inherently carries a risk of selection and verification bias, as imaging modalities were not applied uniformly. Furthermore, 120 patients (24% of those presenting with pathological nipple discharge) were excluded due to missing histopathological confirmation or insufficient follow-up, introducing a risk of partial verification bias. As the availability of an adequate reference standard may have been associated with clinical or imaging findings, these exclusions could have influenced the estimated diagnostic performance and may limit the generalizability of our results. In particular, MRI and ductography were performed only in selected patients, often in equivocal or clinically suspicious cases. Moreover, findings from MRI and ductography could influence the decision to perform ductectomy and thereby the availability of histopathological verification, introducing potential work-up/verification bias. Because these findings also contributed to the indication for ductectomy, which in turn served as the reference standard, incorporation bias may have been present. This is particularly relevant when interpreting the high sensitivity and low specificity observed for ductography.

Thus, diagnostic accuracy metrics reported for these modalities may be influenced by referral bias toward more complex cases and should not be interpreted as estimates for an unselected PND population. Furthermore, as the different imaging modalities were not systematically applied to the same patients, their diagnostic performance cannot be directly compared. Secondly, differential verification was present because malignant and B3 lesions were histologically confirmed, whereas 92 benign cases were classified based on the absence of malignancy during ≥24 months of follow-up.

Patients with ND who did not undergo further diagnostic work-up were not included. Hence, our cohort may overrepresent patients referred to secondary or tertiary care, thereby limiting the generalizability of our findings to the broader population of patients presenting with ND. In addition, PND was defined according to the 2022 ACR Appropriateness Criteria, which require only one of the defining features. Studies applying stricter definitions (e.g., spontaneous, unilateral, single-duct, bloody or serous discharge in combination) may therefore not be directly comparable with our cohort, which also included patients with non-spontaneous or bilateral discharge [52]. Owing to the retrospective design, the individual PND features were not consistently documented for all patients; therefore, a sensitivity analysis restricted to strictly defined PND (spontaneous, unilateral, single-duct discharge) could not be reliably performed. Furthermore, accompanying palpable findings could not be systematically reconstructed across the study cohort, particularly in the B3 and benign groups. However, a sensitivity analysis excluding malignant cases with potentially malignancy-related palpable findings showed only a minor change in the diagnostic performance of the basic imaging work-up.

Moreover, our study lacks long-term follow-up beyond 24 months in some cases, especially for those classified as benign on imaging alone. Therefore, slowly evolving or initially occult malignancies, including low-grade DCIS, cannot be definitively excluded.

## 5. Conclusions

While ultrasound and mammography correctly classify the majority of malignancies as suspicious in patients with PND, negative conventional imaging does not reliably exclude malignancy, and their performance in classifying B3 lesions as suspicious remains limited. In this study, these standard modalities demonstrated high sensitivity and specificity for malignancy but failed to reliably classify lesions of uncertain malignant potential as suspicious. Ductography, although highly sensitive for both malignant and B3 lesions, showed very low specificity, leading to a substantial number of false-positive findings and potentially unnecessary interventions. Given its low specificity, ductography appears to have limited value for reliably differentiating benign from malignant lesions. Its clinical utility may therefore lie primarily in the visualization and localization of the affected ductal system, especially when anatomical mapping is required prior to surgical intervention. MRI, by contrast, offered a more balanced diagnostic profile with acceptable sensitivity and specificity for both lesion categories.

In clinical practice, MRI may be considered as a complementary imaging tool in patients with persistent PND and negative conventional imaging, whereas ductography should primarily be used to localize the affected ductal system, for example prior to surgical duct excision. Future efforts should focus on refining imaging strategies through prospective studies with standardized protocols, integrating novel techniques such as contrast-enhanced ductal imaging or tomosynthesis-based ductography, and evaluating the role of artificial intelligence in improving diagnostic specificity. Optimizing diagnostic pathways will be essential to balance early lesion detection with the avoidance of overtreatment in this challenging patient population.

Beyond evaluating malignancy detection, the present study provides real-world data on the diagnostic performance of conventional and adjunct diagnostic modalities across a broad spectrum of underlying pathologies, including malignant, B3, and benign lesions, thereby illustrating both the strengths and remaining limitations of the diagnostic work-up of PND in routine clinical practice.

## Figures and Tables

**Table 1:** Characteristics of the study cohort.

		*n*	%
Age	mean	51.4 ± 13.9 years	—
Laterality	unilateral	290	74.7
	bilateral	98	25.3
Discharge characteristic	spontaneous	218	56.2
	non-spontaneous	155	40.0
	unknown	15	3.8
Type of discharge	bloody	240	61.9
	clear	114	29.4
	milky	21	5.4
	unknown	13	3.3

**Table 2:** Characteristics of the benign study cohort.

Category	*n*	%
Histological confirmation	104	53.1
– Core needle biopsy	8	7.7
– Stereotactic vacuum-assisted biopsy	3	2.9
– Ductectomy	92	88.5
– MRI-guided vacuum-assisted biopsy	1	1.0
Follow-up ≥ 24 months, no malignancy	92	46.9
Mean age (years)	48.6 ± 13.7	—
Ultrasound performed	196	100
Mammography/tomosynthesis performed	183	93.4
MRI or ductography performed	141	71.9

**Table 3:** Performance of basic diagnostic work-up (ultrasound and mammography).

Group	*n*	Sensitivity	Specificity	PPV	NPV
Malignancy (overall)	40	82.5% (68.1–91.3)	83.5% (77.4–88.2)	52.4% (40.3–64.2)	95.6% (91.2–97.9)
– Invasive breast cancer	14	92.9% (68.5–98.7)	83.5% (77.4–88.2)	30.2% (18.6–45.1)	99.3% (96.4–99.9)
– DCIS	26	76.9% (57.9–89.0)	83.5% (77.4–88.2)	40.0% (27.6–53.8)	96.2% (92.0–98.2)
B3 lesions *	148	33.8% (26.7–41.7)	83.5% (77.4–88.2)	62.5% (51.5–72.3)	60.8% (54.6–66.6)

Legend: Values are percentages with 95% confidence intervals. Analysis sets: malignancy overall *n* = 40 (invasive *n* = 14, DCIS *n* = 26); B3 lesions *n* = 148. Benign reference group *n* = 182. *: For B3 lesions, sensitivity refers to classification as BI-RADS 4/5 and should not be interpreted as lesion-detection sensitivity. B3 lesions visualized but classified as BI-RADS 2 or 3 were considered negative according to the predefined diagnostic performance analysis.

**Table 4:** Imaging findings of B3 lesions other than intraductal papillomas without atypia.

Histo- Pathology	*n*	Ultrasound (BI-RADS)	Mammo- Graphy (BI-RADS)	Ductography	MRI (BI-RADS)	Histological Confirmation
ADH	3	2; 2; 2	2; 2; 2	suspicious; suspicious; suspicious	–; –; –	Duct excision; duct excision; duct excision
ALH	1	4	4	–	–	Core needle biopsy
LCIS	1	3	2	Non-diagnostic (contrast extravasation)	4	Core needle biopsy
Intraductal papilloma with atypia	4	2; 2; 2; 3	2; 2; 2; 3	–; unsuspicious; –; suspicious	4; 4; 4; –	Core needle biopsy; core needle biopsy; core needle biopsy; duct excision
Radial scar	2	2; 4	2; 2	suspicious; Non-diagnostic (contrast extravasation)	–; –	Duct excision; core needle biopsy
Nipple adenoma	2	4; 4	1; 2	–; –	–; –	Punch biopsy; punch biopsy
Intraductal phyllodes tumor	1	2	2	suspicious	–	Duct excision

Legend: ADH, atypical ductal hyperplasia; ALH, atypical lobular hyperplasia; LCIS, lobular carcinoma in situ; MRI, magnetic resonance imaging. “–” indicates that the respective examination was not performed. Ductography with contrast extravasation was considered non-diagnostic. Findings are presented descriptively because of the small number and heterogeneity of lesions; no diagnostic performance measures were calculated.

**Table 5:** Performance of ductography.

Group	*n*	Sensitivity	Specificity	PPV	NPV
Malignancy (overall)	28	89.3% (72.8–96.3)	13.3% (8.2–20.8)	20.3% (14.2–28.3)	83.3% (60.8–94.2)
– Invasive breast cancer	9	66.7% (35.4–87.9)	13.3% (8.2–20.8)	5.8% (2.7–12.0)	83.3% (60.8–94.2)
– DCIS	19	100% (83.2–100)	13.3% (8.2–20.8)	16.2% (10.6–24.0)	100% (79.6–100)
B3 lesions	135	94.8% (89.7–97.5)	13.3% (8.2–20.8)	56.6% (50.1–62.9)	68.2% (47.3–83.6)

Legend: Values are percentages with 95% confidence intervals. Analysis sets: malignancy overall *n* = 28 (invasive *n* = 9, DCIS *n* = 19); B3 lesions *n* = 135. Benign reference group *n* = 113.

**Table 6:** Performance of MRI.

Group	*n*	Sensitivity	Specificity	PPV	NPV
Malignancy (overall)	14	71.4% (45.4–88.3)	90.0% (76.9–96.0)	71.4% (45.4–88.3)	90.0% (76.9–96.0)
– Invasive breast cancer	7	57.1% (25.0–84.2)	90.0% (76.9–96.0)	50.0% (21.5–78.5)	92.3% (79.7–97.3)
– DCIS	7	85.7% (48.7–97.4)	90.0% (76.9–96.0)	60.0% (31.3–83.2)	97.3% (86.2–99.5)
B3 lesions	29	75.9% (57.9–87.8)	90.0% (76.9–96.0)	84.6% (66.5–93.8)	83.7% (70.0–91.9)

Legend: Values are percentages with 95% confidence intervals. Analysis sets: malignancy overall *n* = 14 (invasive *n* = 7, DCIS *n* = 7); B3 lesions *n* = 29. Benign reference group *n* = 40.

**Table 7:** Performance of discharge cytology.

Group	*n*	Sensitivity	Specificity	PPV	NPV
Malignancy (overall)	29	24.1% (12.2–42.1)	98.7% (95.4–99.6)	77.8% (45.3–93.7)	87.3% (81.5–91.5)
– Invasive breast cancer	11	18.2% (5.1–47.7)	98.7% (95.4–99.6)	50.0% (15.0–85.0)	94.4% (89.7–97.0)
– DCIS	18	27.8% (12.5–50.9)	98.7% (95.4–99.6)	71.4% (35.9–91.8)	92.1% (86.9–95.3)
B3 lesions	130	8.5% (4.8–14.5)	98.7% (95.4–99.6)	84.6% (57.8–95.7)	55.9% (50.0–61.7)

Legend: Values are percentages with 95% confidence intervals. Analysis sets: malignancy overall *n* = 29 (invasive *n* = 11, DCIS *n* = 18); B3 lesions *n* = 130. Benign reference group *n* = 153.

## Data Availability

The data supporting the results of this study are available from the corresponding author upon reasonable request due to privacy restrictions.

## Supplementary Materials

The following supporting information can be downloaded at: https://www.mdpi.com/article/10.3390/diagnostics16213530/s1, Table S1: Performance of basic diagnostic work-up (ultrasound and mammography) after exclusion of malignant cases with potentially malignancy-related palpable findings.

## Abbreviations

The following abbreviations are used in this manuscript:

ACR	American College of Radiology
ADH	Atypical ductal hyperplasia
ALH	Atypical lobular hyperplasia
BI-RADS	Breast Imaging Reporting and Data System
B3	Lesions with uncertain malignant potential
CC	Cranio-caudal
CI	Confidence interval
DCIS	Ductal Carcinoma in situ
DBT	Digital breast tomosynthesis
IDP	Intraductal papilloma
LCIS	Lobular carcinoma in situ
MLO	Medio-lateral-oblique
MRI	Magnetic resonance imaging
ND	Nipple discharge
NPV	Negative predictive value
PND	Pathologic nipple discharge
PPV	Positive predictive value
STARD	Standards for Reporting of Diagnostic Accuracy Studies
STROBE	Strengthening the Reporting of Observational Studies in Epidemiology
