# Supplementary material for: Evaluation of the Diagnostic Accuracy of Different Imaging Modalities in Patients with Pathologic Nipple Discharge
Source: Diagnostics (Basel). 2026 Oct 27;16(21):3530. doi: 10.3390/diagnostics16213530 (PMC13761467; doi:10.3390/diagnostics16213530)
Supplement: Supplementary file 1 [file diagnostics-16-03530-s001.zip › diagnostics-4550443-supplementary.pdf]

Sensitivity analysis excluding potentially malignancy-related palpable findings

Among the 40 patients with malignancy included in the basic diagnostic work-up analysis, six had a documented palpable finding for which an association with the subsequently diagnosed malignancy could not be excluded on retrospective review. These included four patients with invasive breast cancer and two patients with DCIS. All six malignancies were correctly identified by ultrasound and/or mammography, although ultrasound was classified as BI-RADS 2 in one case and mammography as BI-RADS 1 in another case. A sensitivity analysis was performed after exclusion of these six patients. Diagnostic performance remained largely unchanged compared with the primary analysis.

Supplementary Table S1 Performance of basic diagnostic work-up (ultrasound and mammography) after exclusion of malignant cases with potentially malignancy-related palpable findings.

Group	n	Sensitivity	Specificity	PPV	NPV
Malignancy (overall)	34	79.4% (63.2–89.7)	83.5% (77.4–88.2)	47.4% (35.0–60.1)	95.6% (91.2–97.9)
– Invasive breast cancer	10	90.0% (59.6–98.2)	83.5% (77.4–88.2)	23.1% (12.6–38.3)	99.3% (96.4–99.9)
– DCIS	24	75.0% (55.1–88.0)	83.5% (77.4–88.2)	37.5% (25.2–51.6)	96.2% (92.0–98.2)

Legend: Values are percentages with 95% confidence intervals calculated using the Wilson score method. Analysis sets: malignancy overall n = 34 (invasive n = 10, DCIS n = 24). Benign reference group n = 182.
